# Nutritional intake and health status of populations and the relationship between diet and oral ulcers: A cross-sectional study based on NHANES data and machine learning predictions

**DOI:** 10.1097/MD.0000000000043383

**Published:** 2025-07-18

**Authors:** Xu Yang, Guangyu Zhang, Qingai Shan

**Affiliations:** a China Aerospace Science & Industry Corporation 731 Hospital, Beijing, China.

**Keywords:** machine learning, NHANES, oral ulcers, risk factors

## Abstract

Oral ulcers are a common oral disease. This study aims to use data from National Health and Nutrition Examination Survey to analyze the related risk factors of oral ulcers and explore health status differences among various populations. The data were derived from National Health and Nutrition Examination Survey, covering various aspects of the health, lifestyle, nutritional status of the U.S. civilian population. Three thousand one hundred twenty-six participants were included in the final analysis, divided into 2 groups: with and without oral ulcers. A standardized questionnaire was used to collect information on age, gender, race, family income, body mass index, diabetes, hyperlipidemia, heart disease, smoking, alcohol consumption. The diagnosis of oral ulcers was based on participants’ self-reported questionnaire results. Statistical analysis was conducted using Statistical Package for the Social Sciences software, including descriptive statistics, Spearman correlation analysis, multiple linear regression, confusion matrix analysis, forest plot analysis, restricted cubic spline regression. Significant differences were found between participants with and without oral ulcers in terms of age, gender, family income, hyperlipidemia, depression, smoking, and alcohol consumption. Age, gender, family income, magnesium, and sodium were important factors related to the incidence of oral ulcers. The model’s accuracy was approximately 72.48%, precision was about 58.26%, recall was about 61.15%, and the F1 score was about 59.57%. The area under the receiver operating characteristic curve was 0.77, indicating that the classifier has a good discriminative ability. There was a significant association between age and the increased risk of oral ulcers, with the risk significantly decreasing with age. Smoking and hypertension had a significant impact on the prediction of oral ulcers, with the model tending to predict the occurrence of oral ulcers in cases with higher levels of smoking and hypertension. Age, gender, family income, hyperlipidemia, depression, smoking, and alcohol consumption are important risk factors for oral ulcers. The model has good predictive ability overall but still has room for improvement in predicting the presence of oral ulcers. There is a significant association between age and the increased risk of oral ulcers, with the risk significantly decreasing with age. Smoking and hypertension have a significant impact on the prediction of oral ulcers.

## 1. Introduction

Oral ulcers are common mucosal lesions of the oral cavity, typically characterized by recurrent, painful sores that significantly impair patients’ ability to eat, speak, and maintain quality of life.^[1]^ Studies have shown that oral ulcers are not only associated with local trauma or infection, but may also reflect underlying systemic health issues such as metabolic disorders, immune dysfunction, or psychological disturbances.^[2–4]^ Epidemiological data indicate that approximately 20% of the global population has experienced oral ulcers, with recurrence rates exceeding 50%.^[5]^ In the United States, data from the National Health and Nutrition Examination Survey (NHANES) reveal marked differences in oral ulcer prevalence across population subgroups, suggesting that a complex interplay of sociodemographic, physiological, and behavioral factors may be involved in its pathogenesis.

Previous research has identified a range of potential contributors to the development of oral ulcers. Gender differences, for instance, are closely linked to mucosal immune responses, with hormonal fluctuations in women potentially increasing susceptibility compared to men.^[6]^ Hyperlipidemia may contribute through low-grade chronic inflammation that impairs mucosal repair mechanisms.^[7]^ Moreover, psychological factors such as depression are associated with immunosuppression and hypothalamic–pituitary–adrenal axis dysregulation, both of which have been implicated in recurrent ulcer formation. Lifestyle behaviors including smoking and alcohol consumption may also promote ulceration by directly irritating the oral mucosa, increasing oxidative stress, or altering the local microbial environment.^[8,9]^ On a socioeconomic level, individuals with lower income may face elevated risk due to inadequate nutrition, poor oral hygiene awareness, and limited access to healthcare services.^[10]^ Nutritionally, imbalances in sodium and magnesium intake have been associated with altered mucosal barrier integrity and may impair tissue repair capacity.^[11]^ Additionally, malnutrition may also increase the risk of oral ulcers, as the lack of necessary nutrients can affect the health and repair capacity of the oral mucosa. Bone loss around teeth may weaken the stability of oral structures, thereby increasing the risk of ulcers. Fluoride drop intake may have a protective effect on oral health, but its direct relationship with oral ulcers still needs further research. Hematologic diseases such as leukemia may lead to abnormal immune function, which in turn increases the incidence of oral ulcers. Irritating foods may directly damage the oral mucosa, triggering or exacerbating ulcers. Poor oral hygiene may lead to the proliferation of bacteria and viruses in the oral cavity, increasing the risk of infection and ulcers.

The innovation of this cross-sectional study lies in its use of nationally representative data from NHANES to systematically evaluate the combined influence of demographic, metabolic, psychological, and behavioral factors on the risk of oral ulcers. Using multivariate linear regression, restricted cubic spline (RCS) modeling, and machine learning approaches, we explored the dynamic nonlinear association between age and ulcer risk (e.g., risk gradually declines after age 30), and constructed a decision boundary model incorporating smoking and hypertension as key predictors. These findings enhance our understanding of the multifactorial etiology of oral ulcers and offer evidence-based insights for public health interventions (particularly in addressing prevention strategies for women), socioeconomically disadvantaged populations, and individuals with metabolic disorders.

## 2. Materials and methods

### 2.1. Data source and study population

This study was based on data from the NHANES, a program conducted by the National Center for Health Statistics in collaboration with the Centers for Disease Control and Prevention. NHANES employs a multistage, stratified probability sampling design to collect nationally representative data on the health, lifestyle, and nutritional status of the noninstitutionalized U.S. population. All participants provided written informed consent, and the study protocol was approved by the National Center for Health Statistics Research Ethics Review Board.

We included adult participants from the NHANES database who had complete data on oral health, demographic characteristics, and dietary intake. A total of 3126 individuals met the inclusion criteria. Participants were categorized into the oral ulcer group or the non-ulcer group based on their responses to oral health-related survey items.

### 2.2. Variable definition and data collection

Based on previous literature and clinical rationale, the following variables were selected as potential covariates: age, sex, race/ethnicity, poverty-income ratio, body mass index (BMI), diabetes, hyperlipidemia, heart disease, depressive status, smoking, and alcohol consumption. Data were extracted from NHANES self-reported questionnaires and laboratory results. Missing data were imputed using interpolation methods in R software.

Oral ulcer status was determined through self-reported responses to 2 NHANES survey questions: “Have you had pain in your mouth during the past 12 months?” and “Do you think you may have gum disease?.” Participants who responded affirmatively to oral pain and denied gum disease were classified as having oral ulcers.

### 2.3. Statistical analysis

All statistical analyses were performed using Statistical Package for the Social Sciences Statistics version 25.0. Participants were divided into oral ulcer and non-ulcer groups. Continuous variables were expressed as mean ± standard deviation and compared using independent samples *t* tests or Mann–Whitney *U* tests, as appropriate. Categorical variables were expressed as counts and percentages, and compared using chi-square tests.

Spearman correlation analysis was conducted to assess the associations between variables. Multivariate linear regression was then used to identify independent risk factors for oral ulcers. To assess nonlinear relationships, RCS regression models were applied. A logistic regression model was constructed to evaluate predictive performance, which was assessed using the receiver operating characteristic (ROC) curve and confusion matrix. All statistical tests were two-sided, and a *P*-value < .05 was considered statistically significant.

## 3. Results

### 3.1. Baseline characteristics

A total of 29,400 NHANES participants were initially screened. After excluding individuals with missing data on key variables such as BMI, hyperlipidemia, diabetes, alcohol consumption, and smoking status, 3126 participants were included in the final analysis. Based on the presence or absence of oral ulcers, participants were divided into the oral ulcer group (n = 1022) and the non-ulcer control group (n = 2103). Significant differences were observed between the 2 groups across several demographic and clinical variables (see Table 1). Compared to those without oral ulcers, the ulcer group had a higher proportion of females (18.2% vs 14.5%, *P* < .001) and low-income participants (18.1% vs 6.8%, *P* < .001). The prevalence of hyperlipidemia (24.9% vs 6.5%, *P* < .001), depression (18.3% vs 12.1%, *P* < .001), smoking (17.8% vs 14.3%, *P* < .001), and alcohol consumption (13.8% vs 6.0%, *P* < .001) was also significantly higher in the ulcer group. Additionally, there were significant differences in age distribution between the 2 groups, with a higher proportion of affected individuals in the 30 to 46 age range (*P* < .01). Moreover, the proportion of malnutrition was higher in the ulcer group (21.4% vs 11.3%, *P* < .001), as was the prevalence of bone loss around teeth (19.3% vs 13.4%, *P* < .001). In terms of oral hygiene, a higher proportion of the ulcer group had poor oral hygiene (32.4% vs 0.3%, *P* < .001). Regarding other health behaviors, a higher proportion of the ulcer group did not take fluoride drops (31.6% vs 1.1%, *P* < .001), and there was a difference in the prevalence of leukemia between the 2 groups (*P* < .001), with a higher proportion of leukemia in the ulcer group (32.7% vs 0.0%). These findings suggest that gender, socioeconomic status, metabolic conditions, psychological health, lifestyle factors, and oral hygiene status may be closely associated with the occurrence of oral ulcers, warranting further investigation into their potential mechanistic roles (Table 1).

**Table 1:** Baseline characteristics.

Variable		Oral ulcer		*P*-value
		No (n = 2103)	Yes (n=1022)	
Age	30–46 years old	484 (15.5%)	278 (8.9%)	<.001
	46–60 years old	598 (19.1%)	324 (10.4%)	
	>60 years old	1021 (32.7%)	420 (13.4%)	
Gender	Male	1065 (34.1%)	454 (14.5%)	<.001
	Female	1038 (33.2%)	568 (18.2%)	
Race	Mexican American	423 (13.5%)	179 (5.7%)	<.001
	Non-Hispanic White	1147 (36.7%)	464 (14.8%)	
	Non-Hispanic Black	533 (17.1%)	379 (12.1%)	
Marital status	Married/living with partner	1263 (40.4%)	532 (17.0%)	<.001
	Never married	211 (6.8%)	151 (4.8%)	
	Widowed/divorced/separated	629 (20.1%)	339 (10.8%)	
Educational	<9th grade	226 (7.2%)	116 (3.7%)	.003
	9th–11th grade or high school	257 (8.2%)	181 (5.8%)	
	High school	551 (17.6%)	254 (8.1%)	
	College or above	1069 (34.2%)	471 (15.1%)	
Income_level	Low income	969 (31.0%)	565 (18.1%)	<.001
	Middle income	366 (11.7%)	245 (7.8%)	
	High income	786 (24.6%)	212 (6.8%)	
BMI		29.9 (26–34.5)	30.3 (26.1–34.9)	.251
Hyperlipidemia	No	1617 (51.7%)	779 (24.9%)	<.001
	Yes	779 (24.9%)	202 (6.5%)	
Diabetes	No	1457 (46.6%)	713 (22.8%)	.783
	Yes	646 (20.7%)	309 (9.9%)	
Heart disease	No	1705 (54.6%)	809 (25.9%)	.205
	Yes	398 (12.7%)	213 (6.8%)	
Protein		1.56 (0.31–6.77)	2.29 (0.32–6.45)	.368
Fiber		0 (0–1.5)	0 (0–1.3)	.814
Magnesium		28.09 ± 40.689	27.87 ± 31.724	.878
Sodium		63 (10–277)	125 (11.75–287.5)	.568
Potassium		137 (67–285)	176 (73–285)	.427
Total fat		0.5 (0.5–5.61)	1.19 (0.06–5.28)	.403
Cholesterol		0 (0–8)	0 (0–35)	.482
Iron		2.0160 ± 4.50608	1.8784 ± 3.76125	.399
Zinc		0.24 (0.07–1.08)	0.36 (0.07–1.105)	.984
Copper		0.092252 ± 0.1312878	0.094607 ± 0.1149474	.625
Vitamin A		0 (0–64)	0 (0–61)	.967
Vitamin B		0.176922 ± 0.3051620	0.169282 ± 0.2510664	.488
Vitamin C		5.8 ± 21.5295	4.942 ± 18.8003	.276
Depression	No depression	1110 (35.5%)	379 (12.1%)	<.001
	Mild depression	410 (13.1%)	184 (5.9%)	
	Severe depression	583 (18.7%)	389 (12.4%)	
Hypertension	No	1268 (41.2%)	612 (19.6%)	<.001
	Yes	817 (26.1%)	363 (11.6%)	
Hemoglobin		29.9 (26–34.5)	29.9 (26–34.5)	<.001
Smoking	Never smoke	1047 (33.5%)	448 (14.3%)	<.001
	Former smoke	628 (20.1%)	244 (7.8%)	
	Current smoke	428 (13.7%)	314 (10.0%)	
Drinking	Never drank	555 (17.8%)	187 (6.0%)	<.001
	Occasionally drank	1089 (34.8%)	299 (9.6%)	
	Regularly drank	144 (4.6%)	37 (1.2%)	
	Heavy drinker	315 (10.1%)	95 (3.0%)	
Malnutrition	No	1624 (52%)	354 (11.3%)	<.001
	Yes	479 (15.3%)	668 (21.4%)	
Bone loss around teeth	No	1786 (57.2%)	419 (13.4%)	.004
	Yes	317 (10.1%)	603 (19.3%)	
Fluoride drop intake	No	649 (20.8%)	987 (31.6%)	<.001
	Yes	1454 (46.5%)	35 (1.1%)	
Leukemia	No	2099 (67.2%)	1 (0.0%)	<.001
	Yes	4 (0.1%)	1021 (32.7%)	
Irritating foods	No	877 (28.1%)	233 (7.5%)	<.001
	Yes	1226 (39.2%)	789 (25.2%)	
Oral hygiene	No	15 (0.5%)	1012 (32.4%)	<.001
	Yes	2088 (66.8%)	10 (0.3%)	

BMI = body mass index.

### 3.2. Forest plot analysis of oral ulcers and included variables

A forest plot was used to visualize the associations between 7 variables (age, sex, hyperlipidemia, depression severity, hypertension, alcohol consumption, and smoking) and the risk of oral ulcers. Each variable was represented by its corresponding odds ratio (OR), 95% confidence interval (CI), and *P*-value.

The analysis revealed associations between all 7 variables and oral ulcer risk. Sex showed a statistically significant association (OR = 1.523, *P* < .001), indicating that females had a 1.523-fold higher risk of developing oral ulcers compared to males. Age demonstrated a nonsignificant trend (OR = 0.899, *P* = .057), suggesting no strong evidence for an age-related effect on ulcer risk.

Hyperlipidemia was significantly associated with increased ulcer risk (OR = 1.311, *P* < .001), indicating that individuals with hyperlipidemia had a 33.1% higher risk compared to those without. Depression severity was also significantly associated with ulcer risk (OR = 1.185, *P* = .005), suggesting an 18.5% increase in risk per unit increase in depression score.

**Figure 1.**
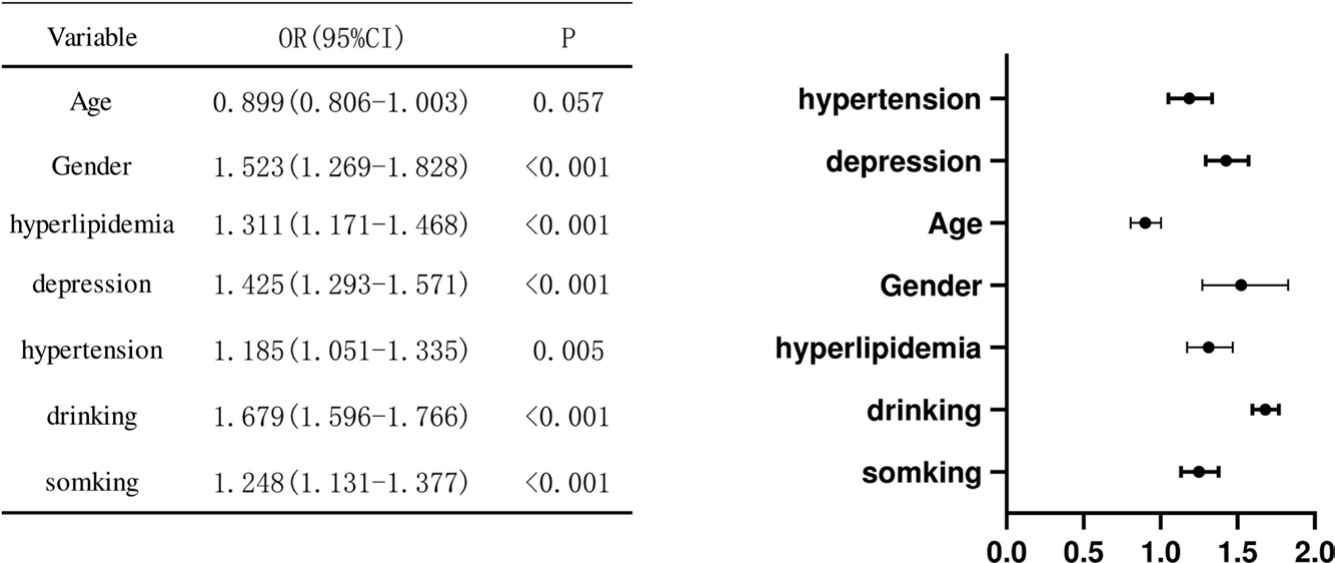
Forest plot of the impact of various variables on oral ulcers. This forest plot illustrates the odds ratios (ORs) and 95% confidence intervals (CIs) for key risk factors associated with the occurrence of oral ulcers. Gender shows a significant trend with an OR of 1.523 (95% CI: 1.269–1.828, *P* < .01). This indicates that the risk of oral ulcers in females is 1.523 times higher than in males. Age shows a nonsignificant trend with an OR of 0.899 (95% CI: 0.806–1.003, *P* = .057). This suggests that there is no significant association between age and the risk of oral ulcers. Hyperlipidemia shows a significant trend with an OR of 1.311 (95% CI: 1.171–1.468, *P* < .001). This means that the risk of oral ulcers in individuals with hyperlipidemia is 33.1% higher than in those without hyperlipidemia. Depression level also shows a significant trend with an OR of 1.185 (95% CI: 1.051–1.335, *P* = .005). This indicates that for every unit increase in depression level, the risk of oral ulcers increases by 18.5%. Alcohol consumption shows a significant trend with an OR of 1.679 (95% CI: 1.596–1.766, *P* < .001). This means that for every unit increase in alcohol consumption, the risk of oral ulcers increases by 67.9%. Smoking also shows a significant trend with an OR of 1.248 (95% CI: 1.131–1.377, *P* < .001). This indicates that for every unit increase in smoking, the risk of oral ulcers increases by 24.8%. Overall, this forest plot helps us understand the relationship between various factors and the risk of oral ulcers. It is important to note that these results do not imply causation but rather indicate the correlation between risk factors and the disease. CI = confidence interval.

Alcohol consumption showed a strong positive association (OR = 1.679, *P* < .001), indicating that each unit increase in alcohol intake was associated with a 67.9% increase in the risk of oral ulcers. Similarly, smoking was significantly associated with higher risk (OR = 1.248, *P* < .001), suggesting a 24.8% increased risk per unit of smoking exposure (Fig. 1).

### 3.3. Multivariate linear regression analysis of risk factors for oral ulcers

A multivariate linear regression model was used to analyze the potential risk factors associated with oral ulcers. The results indicated that age, sex, hyperlipidemia, diabetes, depression, hypertension, smoking, and alcohol consumption were significantly associated with the occurrence of oral ulcers.

Specifically, age was inversely associated with ulcer risk (*B* = ‐0.021, *P* = .019), suggesting that for each unit increase in age, the likelihood of developing oral ulcers decreased by 0.021 units. Sex showed a significant effect (*B* = 0.075, *P* < .001), indicating that females had a 1.523-fold higher risk of oral ulcers compared to males. Hyperlipidemia was positively associated with ulcer risk (*B* = 0.033, *P* < .001), as individuals with hyperlipidemia had a higher likelihood of experiencing oral ulcers than those without. Diabetes also showed a significant association (*B* = 0.037, *P* = .025), indicating an elevated risk among diabetic individuals.

Other variables, such as BMI and hypertension, did not show statistically significant associations in this model. The variance inflation factors for most variables ranged from 1 to 1.5, indicating that multicollinearity was not a concern in the regression analysis (Table 2).

**Table 2:** Multiple linear regression model for oral ulcer.

	B	SE	*t*	*P*-value	VIF
Age	‐0.021	0.009	-2.342	.019	1.078
Gender	0.075	0.015	5.130	<.001	1.047
BMI	0.001	0.001	1.308	.191	1.105
Hyperlipidemia	0.033	0.007	4.409	<.001	1.032
Diabetes	0.037	0.016	2.250	.025	1.109
Depression	0.036	0.006	5.856	<.001	1.168
Hypertension	0.009	0.007	1.294	.196	1.125
Smoking	0.097	0.004	27.417	<.001	1.166
Drinking	0.038	0.008	4.767	<.001	1.046

BMI = body mass index.

### 3.4. Correlation analysis of factors associated with oral ulcers

Correlation analysis revealed several variables significantly associated with the occurrence of oral ulcers. Age showed a significant negative correlation with oral ulcer risk (*r* = ‐0.069, *P* < .001), indicating that the likelihood of developing oral ulcers decreases with increasing age. Sex was positively correlated with ulcer occurrence (*R* = 0.058, *P* < .001), suggesting that females are at a higher risk compared to males. Household income was negatively correlated with oral ulcer risk (*r* = ‐0.127, *P* < .001), indicating that individuals with higher income levels had a lower likelihood of developing ulcers.

Dietary fiber intake was positively associated with oral ulcer occurrence (*R* = 0.039, *P* = .031), implying that each unit increase in fiber intake corresponded to a 0.039-fold increase in ulcer risk. Similarly, magnesium intake showed a significant positive correlation (*R* = 0.045, *P* = .011), as did sodium intake (*R* = 0.051, *P* = .004), indicating that increased intake of these nutrients was associated with higher ulcer risk.

Other variables, such as hypertension and heart disease, did not show statistically significant correlations with oral ulcers (*P* > .05). It is worth noting that although some variables were not significantly correlated, they may still influence ulcer development to some extent, which warrants further investigation.

Overall, age, sex, income level, magnesium intake, and sodium intake were identified as key variables significantly correlated with the occurrence of oral ulcers. Future studies are needed to explore the underlying mechanisms behind these associations (Table 3).

**Table 3:** The table shows the correlation analysis of oral ulcers.

Variable	Correlation coefficient	*P*-value
Age	‐0.069**	<.001
Gender	0.058**	<.001
Income_level	‐0.127**	<.001
Fiber	0.039*	.031
Magnesium	0.045*	.011
Sodium	0.051**	.004
Depression	0.188**	<.001
Drinking	0.323**	<.001
Hypertension	0.029	.103
Heart disease	0.023	.205

* When the confidence level (double test) is 0.05, the correlation is significant.

** When the confidence level (double test) is 0.001, the correlation is significant.

### 3.5. Confusion matrix analysis of oral ulcers

The confusion matrix is a key evaluation tool used to compare the predicted outcomes of a classification model with actual observed labels. In this study, we analyzed the confusion matrix to assess the model’s performance in predicting oral ulcer status.

The results showed that the model achieved an overall accuracy of approximately 72.48%, indicating a relatively high level of prediction accuracy. The precision was about 58.26%, meaning that among all samples predicted as positive (oral ulcer cases), 58.26% were correctly classified. The recall (sensitivity) was approximately 61.15%, indicating that a majority of true positive cases were successfully identified by the model. The F1 score, which represents the harmonic mean of precision and recall, was calculated to be around 59.57%.

**Figure 2.**
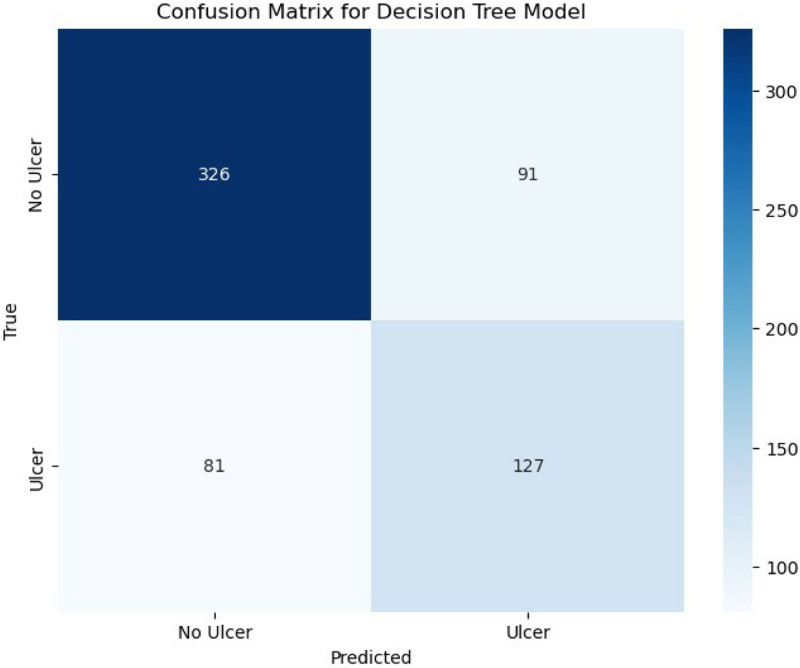
Confusion matrix. The horizontal axis (Predicted): represents the categories predicted by the model. “No Ulcer”: the model predicts that there is no oral ulcer. “Ulcer”: the model predicts that there is an oral ulcer. The vertical axis (True): represents the actual category labels. “No Ulcer”: there is actually no oral ulcer. “Ulcer”: there is actually an oral ulcer. True Negative (TN): 326, indicating the number of samples that the model correctly predicted as having no oral ulcer. False Positive (FP): 91, indicating the number of samples that the model incorrectly predicted as having an oral ulcer. False Negative (FN): 81, indicating the number of samples that the model incorrectly predicted as having no oral ulcer. True Positive (TP): 127, indicating the number of samples that the model correctly predicted as having an oral ulcer. This confusion matrix can help you more intuitively understand the model’s predictive performance, especially for positive (with oral ulcer) and negative (without oral ulcer) samples.

In summary, these metrics suggest that the model demonstrates good overall predictive capability. However, there is room for improvement in correctly identifying positive cases, particularly in enhancing both precision and recall. To further improve model performance, it is recommended to fine-tune model parameters and optimize the underlying algorithm settings (Fig. 2).

### 3.6. Model-based prediction of oral ulcers

In this study, we explored the association between oral ulcers and the included variables by developing a predictive model. The model demonstrated that these factors could collectively influence the risk of developing oral ulcers to a considerable extent.

**Figure 3.**
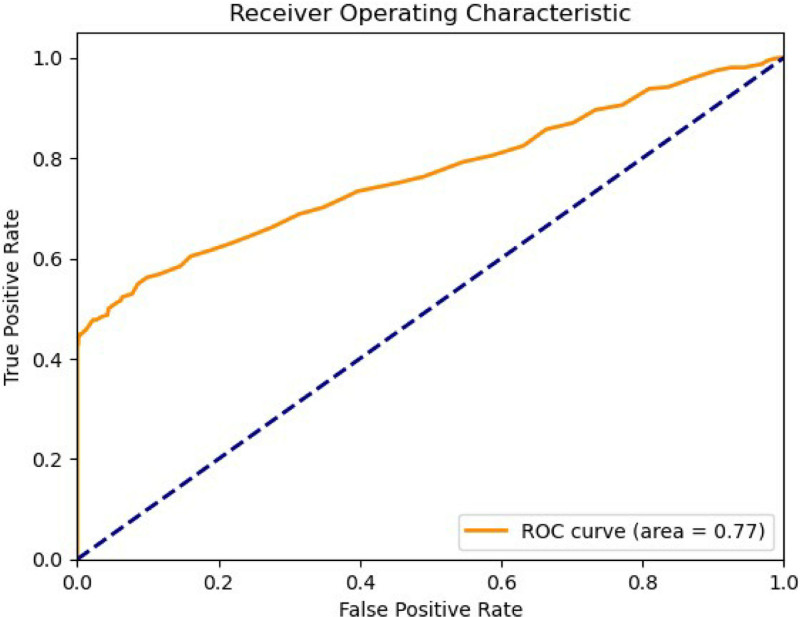
Receiver operating characteristic (ROC) curve. This graph depicts the ROC curve, which is used to assess the performance of a classification model. Horizontal axis: represents the false positive rate (FPR), which is the proportion of false alarms or incorrect positive predictions. Vertical axis: represents the true positive rate (TPR), which is the proportion of correctly predicted positives. The orange line in the graph represents the ROC curve, and the area under this curve (area under curve, AUC) is 0.77. The AUC value ranges from 0 to 1, with higher values indicating a stronger discriminative ability of the model. Here, an AUC of 0.77 suggests that the model performs fairly well in distinguishing between different categories. The blue dashed line is the diagonal, representing the performance of random guessing, with an AUC of 0.5. Therefore, this graph illustrates the accuracy of the studied model in predicting oral ulcers.

Specifically, the ROC curve was used to evaluate the performance of the classifier. The *x*-axis represents the false positive rate, while the *y*-axis denotes the true positive rate. In the ROC plot, the orange solid line represents the ROC curve with an area under the curve of 0.77, indicating good discriminatory power of the model. The blue dashed line represents the line of random guessing, with an area under the curve of 0.5, serving as a baseline reference. The closer the ROC curve is to the top-left corner of the graph, the better the classification performance (Fig. 3).

### 3.7. RCS regression analysis of the nonlinear association between age and oral ulcers

RCS regression was performed to explore the potential nonlinear relationship between age and the risk of oral ulcers after adjusting for relevant covariates. The resulting plot illustrates the estimated hazard ratios with corresponding 95% CIs across different age levels.

The analysis demonstrated a significant association between age and the risk of oral ulcers. As shown in the figure, the hazard ratio gradually declined with increasing age, indicating that older individuals had a lower risk of developing oral ulcers. Specifically, the relative risk appeared higher around age 30, but steadily decreased as age increased, leveling off at approximately 60 years. Notably, the risk dropped more sharply after age 50, which may be related to physiological changes, alterations in immune function, or shifts in lifestyle habits.

**Figure 4.**
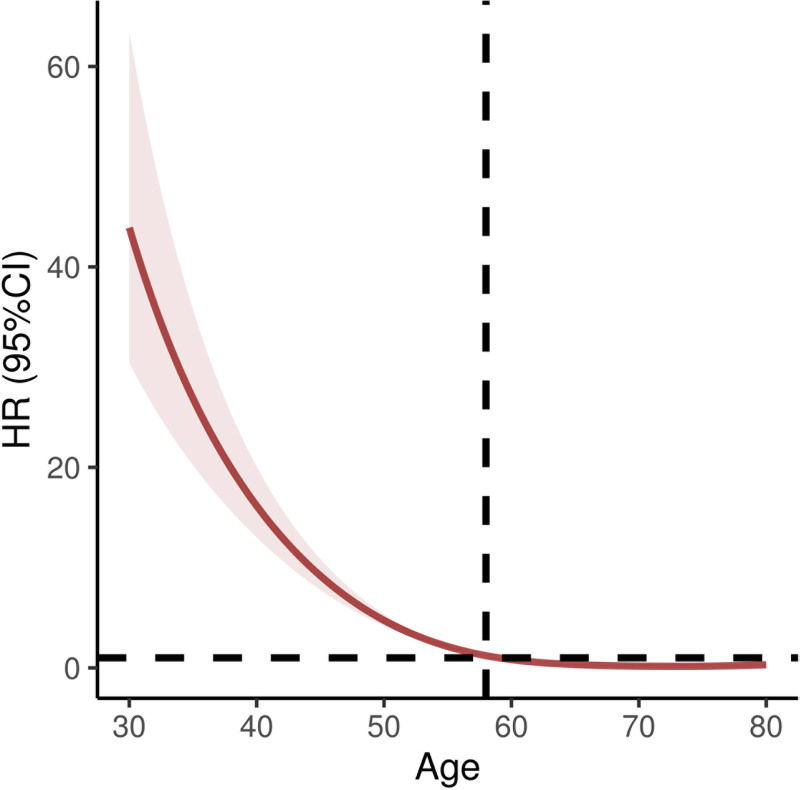
Relationship between age and risk of oral ulcers. Horizontal axis (*X*-axis): represents age, with values ranging from 30 to 80. This could be a scale used to assess the age of individuals. Vertical axis (*Y*-axis): Indicates the hazard ratio (HR) and its 95% confidence interval (CI). Red line: illustrates the trend of the hazard ratio as age increases. This line helps us visually understand the relationship between age and the risk of oral ulcers. Shaded area: represents the 95% confidence interval, depicting the range of reliability of the estimate. A narrower confidence interval indicates a more precise estimate, while a wider interval suggests greater uncertainty. According to the content of the chart, as age increases, the hazard ratio gradually decreases. This suggests that older age may be associated with a reduced risk of oral ulcers. This association might imply that more attention should be paid to the screening and preventive measures for oral ulcers. It should be noted that while the chart suggests a relationship between age and the risk of oral ulcers, it does not establish a causal link. Further research would be needed to explore the underlying mechanisms that could explain this relationship.

The shaded red area in the graph represents the 95% CI, reflecting the precision of the estimated risk. A narrower interval suggests greater reliability. These findings highlight the importance of heightened oral health awareness and preventive care among younger adults, particularly those aged 30 to 40 years. Although older adults exhibit a lower overall risk, maintaining proper oral hygiene remains essential to prevent other oral health issues (Fig. 4).

### 3.8. Decision boundary analysis for predicting oral ulcers

**Figure 5.**
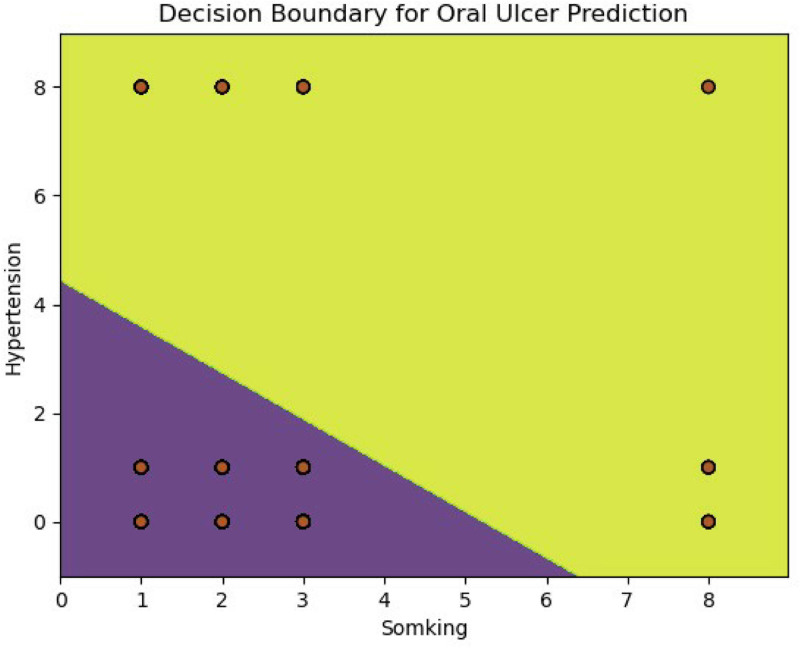
Decision boundary for oral ulcer prediction. *X*-axis: labeled as “Smoking,” ranging from 0 to 8. *Y*-axis: labeled as “Hypertension,” ranging from 0 to 8. Color regions: yellow region indicates the area predicted as non-oral ulcers. Purple region: indicates the area predicted as oral ulcers. Data points: brown circles: represent samples that actually have oral ulcers. Light blue circles: represent samples that do not have oral ulcers. Decision boundary: the decision boundary is a diagonal line that separates the purple and yellow regions. This line represents the threshold for the model’s prediction of the occurrence of oral ulcers. Data distribution: most of the brown circles (samples that actually have oral ulcers) are located in the lower-left purple region, indicating that under these conditions, the model is more inclined to predict the presence of oral ulcers. The light blue circles (samples without oral ulcers) are mostly located in the upper-right yellow region, indicating that under these conditions, the model is more inclined to predict non-oral ulcers. Summary: this chart visually demonstrates how the logistic regression model predicts the likelihood of oral ulcers based on the 2 features of smoking and hypertension. The purple region indicates a high probability of having oral ulcers, while the yellow region indicates a low probability. The decision boundary clearly delineates the boundary between these 2 regions.

The decision boundary derived from the logistic regression model provided a clear visualization of how smoking and hypertension contribute to oral ulcer risk stratification. As shown in the boundary plot, the model effectively distinguishes between participants with and without oral ulcers, suggesting robust classification performance (Fig. 5).

Smoking demonstrated a strong predictive effect, with individuals exhibiting higher smoking levels (leftward along the *x*-axis) more likely to be classified as having oral ulcers. Hypertension also contributed to the model, albeit to a lesser extent; participants with higher blood pressure levels (indicated lower on the y-axis) tended to fall within the high-risk region.

The model’s prediction pattern aligned with biological plausibility: both smoking and elevated blood pressure are known to impair mucosal integrity and modulate inflammatory responses, which may predispose individuals to ulcer formation. The distribution of actual ulcer cases (depicted as brown circles) was mainly concentrated in the lower-left quadrant of the graph, while non-ulcer cases (light blue circles) were mostly located in the upper-right quadrant. This spatial segregation further supports the model’s discriminatory ability.

The visualization also highlighted risk regions, where the purple area denotes high predicted probabilities of oral ulcers, and the yellow area indicates low probabilities. The clear separation between these regions offers an intuitive tool for identifying high-risk populations.

In summary, the decision boundary analysis revealed that smoking and hypertension jointly delineate a meaningful threshold for oral ulcer risk. Although promising, future research should incorporate additional predictors, validate the model in external populations, and compare performance across alternative machine learning algorithms to ensure broader applicability and clinical utility.

## 4. Discussion

This study utilized data from the NHANES to analyze the risk factors associated with oral ulcers and to explore health disparities across different population subgroups. The findings indicate that age, sex, household income, hyperlipidemia, depression, smoking, and alcohol consumption are significant risk factors for oral ulcer occurrence. While the predictive model demonstrated overall good performance, there remains room for improvement in accurately identifying individuals with oral ulcers. A notable inverse association was observed between age and the risk of developing oral ulcers, with the risk significantly decreasing as age increases.^[12]^ Additionally, smoking and hypertension were found to have significant predictive value for oral ulcer development.^[13]^

The observed decline in oral ulcer risk with increasing age may be attributed to various factors, including lifestyle modifications, immunological changes, and improved oral hygiene among older adults. The concept of the “senescence-associated secretory phenotype” in aging immune systems may contribute to a state of chronic low-grade inflammation, which paradoxically enhances mucosal repair mechanisms.^[14]^ Furthermore, dietary changes such as reduced intake of spicy or irritant foods and the frequent use of antioxidant medications among elderly individuals may offer additional protective effects against ulcer formation.^[15]^

Women were found to have a significantly higher risk of developing oral ulcers compared to men. This difference may be attributed to hormonal fluctuations and variations in immune response. Specifically, fluctuations in estrogen and progesterone levels in females may induce vasoconstriction and inflammatory responses in the oral mucosa, thereby increasing the likelihood of ulcer formation. This risk tends to peak during the luteal phase of the menstrual cycle and the perimenopausal period. These findings suggest that estrogen fluctuations may influence local immune responses by modulating the expression of Toll-like receptors in oral epithelial cells.^[16]^

Low-income individuals were also observed to have a significantly elevated risk of oral ulcers. This may be due to inadequate nutritional intake, poor oral hygiene practices, and limited access to healthcare services.^[17]^ Additionally, individuals with hyperlipidemia were found to have an increased risk, suggesting that lipid metabolism disorders may impair mucosal healing through inflammatory pathways. Depression was associated with an increased risk of oral ulcers, likely mediated by stress-induced immune dysregulation and oxidative stress.^[18]^ Both smoking and alcohol consumption were also significantly linked to a higher risk of oral ulcers, possibly due to the direct irritant and damaging effects of tobacco and alcohol on the oral mucosal lining.

Dietary factors such as increased sodium and magnesium intake were positively associated with the risk of oral ulcers, suggesting that nutritional imbalance may indirectly contribute to ulcer formation through disruption of mucosal barrier function. A high-sodium diet can lead to dehydration of oral mucosal cells, compromising the integrity of the mucosal barrier and increasing sensitivity to irritants, thereby elevating the risk of oral ulceration.^[19]^ Magnesium, as a cofactor in numerous enzymatic reactions, plays a critical role in cellular metabolism and immune function. Magnesium deficiency can impair oral mucosal cell function and reduce barrier integrity, thereby increasing susceptibility to ulcer formation.^[20]^ A diverse diet that ensures adequate intake of vitamins, minerals, and dietary fiber is essential for maintaining oral mucosal health and preventing ulceration. Limiting the consumption of processed foods, pickled products, and salt-based condiments can help reduce sodium intake. Additionally, magnesium supplementation through diet or supplements may help prevent deficiency. Magnesium-rich foods include leafy green vegetables, nuts, seeds, whole grains, and legumes. Maintaining a balanced diet, minimizing high-sodium food intake, and ensuring sufficient micronutrient consumption are key strategies for the prevention of oral ulcers.

To effectively prevent the occurrence of oral ulcers, it is essential to implement targeted oral health education tailored to different population groups, thereby enhancing public awareness of the etiology, clinical manifestations, and prevention and treatment strategies of the disease. A balanced diet should be promoted, ensuring adequate intake of micronutrients such as vitamin B12, folic acid, iron, and zinc, which are closely related to mucosal repair. At the same time, consumption of irritating foods (including spicy, acidic, or overly hot items) should be minimized to reduce mucosal injury. Promoting good oral hygiene practices, such as brushing teeth twice daily, rinsing the mouth after meals, and using dental floss, is also crucial. In addition, interventions targeting smoking and alcohol consumption should be strengthened to reduce their harmful effects on the oral mucosa. Attention should also be given to psychological support for individuals under high stress or with depressive symptoms to improve overall immune function. The findings of this study provide evidence-based support for public health policies, especially for health education, nutritional improvement, and systematic oral care targeting high-risk groups such as women, individuals with low income, and those with metabolic abnormalities. Clinicians may also utilize these results to identify high-risk individuals and guide personalized prevention and follow-up strategies.

### 4.1. Limitations

Data limitations: Based on the US population data from NHANES, the conclusions are limited to other races/regions. Nutrient intake data rely on self-report and may be subject to recall bias. The mechanism exploration of future research directions, combined with molecular biology methods, and analyze the role of inflammatory factors and nutrient metabolism pathways in oral ulcer. Refining the variable analysis, focusing on the dose-response relationship between vitamin/mineral deficiency and oral ulcer, can be further verified by RCS curve. A dynamic prediction model should be constructed to verify the long-term effect in the follow-up population.

## 5. Conclusion

This study, based on the NHANES database, identified multiple significant risk factors for oral ulcers, including age, sex, household income, hyperlipidemia, depression, smoking, and alcohol consumption. The constructed prediction model demonstrated good discriminative performance, offering valuable support for screening and preventive strategies among high-risk populations. Future studies should further investigate the pathophysiological mechanisms of oral ulcers, optimize prediction models, and evaluate the effectiveness of various interventions, with the ultimate goal of reducing disease incidence and improving patient quality of life.

## Author contributions

**Data curation:** Qingai Shan.

**Formal analysis:** Qingai Shan.

**Investigation:** Xu Yang, Guangyu Zhang.

**Methodology:** Qingai Shan.

**Software:** Guangyu Zhang, Qingai Shan.

**Writing – original draft:** Xu Yang, Qingai Shan.
